# Exposure to Terrorism, Post-Traumatic Stress Disorder and University Teachers’ Performance: Underpinning the Role of Social Support

**DOI:** 10.3390/bs13060517

**Published:** 2023-06-19

**Authors:** Farida Saleem, Muhammad Imran Malik

**Affiliations:** 1 Department of Management, College of Business Administration, Prince Sultan University, Riyadh 11586, Saudi Arabia; 2 Department of Management Sciences, COMSATS University Islamabad, Attock Campus, Attock 43600, Pakistan

**Keywords:** terrorism, post traumatic stress disorder, social support, university, employees’ performance

## Abstract

This study aims to investigate the impact of exposure to terrorism on post-traumatic stress disorder and employee performance, and to determine whether social support acts as a boundary condition that can attenuate the adverse effects of PTSD on employee performance. The study used a cross-sectional sample of 178 university teachers who had experienced a terrorist attack. Data was collected using closed-ended questionnaires and analyzed using PROCESS Macro). The results found a negative and significant relationship between exposure to terrorism, post-traumatic disorder, and employees’ performance. Moreover, it was also found that social support helps attenuate the negative impact of PTSD on performance levels. This study adds to the existing body of knowledge by exploring the relationship between terrorism exposure, PTSD, employee performance, and the potential mitigating effects of social support.

## 1. Introduction

Environmental changes, including exposure to terrorism, are a growing concern for employees worldwide. This has increased the need to examine the impact of terrorism on employee work outcomes. Gürsoy and Chi [1] found that employees directly exposed to terrorism experienced a significant decline in job performance. Exposure to terrorism can have a profound negative impact on university employee performance. Studies have shown that such exposure can lead to significant levels of stress and anxiety, which can negatively affect cognitive function, job satisfaction, and overall job performance [2,3]. Prior research has found that employees who live in areas that are frequently targeted by terrorism are more likely to experience symptoms of post-traumatic stress disorder (PTSD), including intrusive thoughts, avoidance behaviors, and hyperarousal [4]. These symptoms can negatively impact job performance by decreasing focus and motivation and increasing absenteeism and turnover [3]. PTSD disorder leads to occupational instability and can severely impair the daily lives of those who experience it [5,6].

Moreover, individuals with a sound psychological state tend to have a higher probability of participating in and fulfilling jobs and can enjoy a better standard of living [7]. However, vulnerable individuals may lack adequate social support, making it harder to handle stress. It can result in them isolating themselves from essential members of society, causing occupational alienation. To ensure that teachers can effectively perform their jobs and provide students with a supportive learning environment, it is critical to address the issue of PTSD among teachers, as it significantly impacts work performance and overall well-being [8]. The current study posits that social support is an important boundary condition that can attenuate the negative relationship between PTSD and job performance among university teachers. Exposure to terrorism can result in Post-Traumatic Stress Disorder (PTSD), negatively affecting employee performance. Social support (SS) can reduce symptoms of PTSD and improve work performance. Social support helps individuals reframe their thoughts and behaviors, reducing the impact of trauma on their daily lives. Employers can encourage employees to seek social support, promoting resilience and mitigating the adverse effects of exposure to terrorism on their work performance.

This research presents a holistic model of terrorism exposure and employee performance, taking social support as a boundary condition and post-traumatic stress disorder as an explanatory mechanism. We have contributed to the literature in two significant ways. Firstly, we have tried to explain the means through which terrorism exposure negatively affects the performance of employees and how this negative effect can be managed with the help of social support. Secondly, we have used data from university teachers from a developing economy, which will help generalize the findings from developed and Western economies.

The findings of this study suggest that interventions aimed at improving social support among university teachers may help to prevent or mitigate the negative impact of PTSD on their job performance. It is important for employers to be aware of the potential impact of terrorism exposure and PTSD on their employees’ work performance and to provide appropriate support and accommodations. Universities need to provide resources and support to their faculty members to help them cope with the potential exposure to traumatic events and reduce the risk of developing PTSD. These findings also highlight the need for organizations to implement measures to support employees who are affected by terrorism and to mitigate its impact on their performance.

## 2. Literature Review

### 2.1. Exposure to Terrorism and Employee Performance

Gürsoy and Chi [1] used a sample of Turkish employees and revealed that those directly affected by terrorism reported lower levels of job satisfaction, increased absenteeism, and decreased organizational commitment. Similarly, Bader and Berg [9] examined the impact of terrorism on the performance of employees in India. They found that exposure to terrorism was associated with lower job satisfaction and increased stress levels, leading to decreased employee performance. The authors concluded that the long-term impact of terrorism on employee performance is a significant concern for organizations and policymakers. Additionally, Ndubisi and Hartel [10] explored the relationship between exposure to terrorism and job performance among hospitality employees in Australia’s hospitality industry. The findings showed that employees who were exposed to terrorism reported lower job satisfaction, increased anxiety, and decreased job performance compared to those who were not exposed.

The available literature suggests that exposure to terrorism can have a negative impact on employee performance. Employees who are directly exposed to terrorism report lower job satisfaction, increased stress levels, and decreased organizational commitment, leading to reduced job performance. Hence based on the above findings from the literature, the following hypothesis is proposed.

**Hypothesis** **1.** *Exposure to Terrorism has a negative impact on the performance of university teachers*.

### 2.2. Exposure to Terrorism and Post-Traumatic Stress Disorder

Exposure to terrorism and the aftermath of such events can significantly impact an individual’s mental health. University teachers, who are often at the forefront of societal recovery and reconstruction following acts of terrorism, are no exception. Research has shown that exposure to terrorism can lead to PTSD in university teachers, with several studies finding that rates of PTSD can be as high as 20–30% among those who have been directly exposed to acts of terrorism [11]. The symptoms of PTSD can have a profound effect on an individual’s life, and for university teachers, this can have implications for their work and personal life [12]. PTSD can lead to feelings of anxiety, irritability, and depression, which can make it difficult for teachers to perform their job effectively and engage with students [8].

In addition, research has shown that exposure to terrorism can also impact an individual’s sense of security and overall quality of life [13]. The high rates of PTSD among university teachers [14] who have been exposed to terrorism is essential to address this issue through effective prevention and treatment programs [15]. This may involve providing teachers with access to mental health services and support groups, and also implementing measures to improve their sense of safety and security in the workplace. Based on the above findings, it is assumed that exposure to terrorism can significantly impact university teachers, leading to PTSD and other mental health issues.

**Hypothesis** **2.** *Exposure to terrorism positively impacts Post Traumatic Stress Disorder among university teachers*.

### 2.3. Post-Traumatic Stress Disorder and Employee Performance

Post-Traumatic Stress Disorder (PTSD) is a mental health condition that can develop after experiencing or witnessing a traumatic event. It can cause symptoms such as intrusive thoughts, flashbacks, avoidance behaviors, and increased anxiety and arousal. PTSD can significantly impact an individual’s daily life and functioning, including work performance [5,6,12]. Bakker et al. [16] investigated the relationship between PTSD and job performance among university teachers. The study found that teachers with PTSD reported lower levels of job satisfaction, higher levels of burnout, and a decrease in their overall work performance compared to teachers without PTSD. The teachers with PTSD also reported increased absenteeism and higher levels of emotional exhaustion.

Further, Micali et al. [17] found that teachers with PTSD showed lower levels of job performance, as measured by their ability to handle stress and overall job performance ratings. In addition, the study found that teachers with PTSD were more likely to experience feelings of isolation and were less likely to seek support from either colleagues or superiors. Therefore, based on the above findings, it is hypothesized that PTSD can significantly negatively impact the work performance of university teachers.

**Hypothesis** **3.** *Post-Traumatic Stress Disorder negatively affects the performance of university teachers*.

### 2.4. Post-Traumatic Stress Disorder as a Mediator between TE and EP

Exposure to terrorism can be one such traumatic event that can lead to PTSD, which in turn can significantly impact an individual’s work performance. Existing literature has shown that exposure to terrorism can lead to the development of PTSD. For example, Schiff [18] found that individuals who had experienced terrorism had higher rates of PTSD than those who had not. Similarly, a study by Norris et al. [19] found that individuals who had experienced terrorism-related trauma had higher rates of PTSD than those who had experienced other types of trauma.

PTSD has a significant impact on an individual’s work performance. Bryant et al. [20] found that PTSD symptoms were associated with lower job satisfaction and increased work-related stress. Another study by Hoge et al. [21] found that PTSD was associated with decreased work performance and increased absenteeism. PTSD can mediate the relationship between terrorism exposure and employee performance. A study by Schiff [18] found that PTSD mediated the relationship between terrorism exposure and work productivity. Similarly, a study by Pargament et al. [22] found that PTSD mediated the relationship between exposure to terrorism and job satisfaction.

There are several potential mechanisms through which PTSD can impact work performance. PTSD can lead to intrusive thoughts and flashbacks, distracting individuals from work. PTSD can also lead to avoidance behaviors, such as avoiding situations that remind individuals of their traumatic events, leading to decreased work performance. Additionally, PTSD can lead to emotional numbness and reduced motivation, impacting work performance. This study posits that exposure to terrorism can lead to PTSD, which can significantly affect an individual’s work performance. PTSD mediates the relationship between terrorism exposure and employee performance, and there are several potential mechanisms through which PTSD can impact work performance.

**Hypothesis** **4.** *Post-Traumatic Stress Disorder mediates the relationship between terrorism exposure and the performance of university teachers*.

### 2.5. Social Support as a Moderator between PTSD and EP

Social support comprises the resources that individuals receive from their social networks, including emotional, informational, and instrumental support [23]. In universities, social support comes from colleagues, supervisors, and students, and it impacts a teacher’s job satisfaction, well-being, and performance. Prior research findings suggest that social support positively impacts university teachers’ performance. For example, a study by Chi et al [24] found that social support from colleagues was positively associated with teaching effectiveness and well being among teachers. Similarly, Liu et al [25] found that social support from colleagues and supervisors was positively related to job satisfaction and performance among faculty members.

Social support also buffers the negative effects of stress and burnout on university teachers’ performance [26]. This highlights the importance of fostering a supportive work environment that encourages social support among colleagues and supervisors. Social support significantly impacted university teachers’ job satisfaction, well-being, and performance, emphasizing the importance of creating a supportive work environment to promote positive outcomes. University teachers are particularly vulnerable to PTSD due to the potential for exposure to traumatic events, such as student suicides, violence on campus, and natural disasters. PTSD negatively affects teachers’ performance and well-being. Social support is a potential moderator between PTSD and work-related outcomes such as job performance [27]. Liu et al. [28] suggested that teachers with PTSD can suffer from impaired thinking and experience decreased performance. They emphasized the importance of social support in mitigating the adverse effects of PTSD among university teachers. The study suggests that interventions aimed at improving social support among university teachers may help to prevent or reduce the negative impact of PTSD on job performance.

Social support is the assistance and encouragement provided by colleagues, supervisors, and family members to individuals in various situations, including the workplace. Social support has a positive impact on employee performance. Bakker et al. [16] found that social support from colleagues and supervisors positively relates to job performance. Similarly, Eisenberger et al. [29] found that social support from family members positively predicts job satisfaction and performance. In addition, social support can also buffer the adverse effects of stress on employee performance. Kim et al. [30] found that social support from supervisors and coworkers helps mitigate the negative impact of job stress on employee performance. These findings suggest that social support is essential when managing organizational employee performance. Hence, based on prior literature findings, the following hypothesis is proposed.

**Hypothesis** **5.** *Social Support attenuates the negative impact of PTSD on the performance of university teachers*.

**Figure 1:**
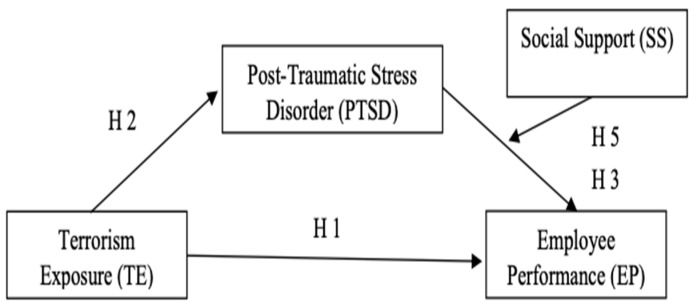
Theoretical Framework.

Examining the impact of exposure to terrorism on post-traumatic stress disorder (PTSD) and employee performance is crucial due to the pervasive and long-lasting effects of terrorism on individuals and communities [19]. Understanding the relationship between terrorism exposure, PTSD, and employee performance can help organizations develop effective interventions and support strategies for employees who have been affected by terrorism [31]. Additionally, exploring the role of social support as a potential boundary condition can provide insights into how supportive environments may buffer the negative effects of PTSD on employee performance [3]. This research can inform organizational policies and practices to promote employee well-being and resilience in the aftermath of terrorism. The theoretical/research framework is presented in Figure 1.

## 3. Methodology

### 3.1. Population and Sampling

The study data was gathered two months after the terrorist attack on a University in Pakistan. The study got approval from the ethics committee for ethical considerations. The research was performed per the Declaration of Helsinki and its later amendments and comparable ethical standards. Informed consent was obtained from each participant before data collection. All study participants will be given complete information about the study’s aims and the data’s confidentiality. A total of 178 responses from university teachers based on purposive sampling were collected. Our inclusion criterion was the teachers’ presence during the incident. The majority of the respondents were male (65%). Respondents aged between 26 to 55 years with educational qualifications ranging from undergraduate to doctorate degrees. The participants were not provided with any reward for completing the survey questionnaire. However, the participation was voluntary, and respondents were allowed to retreat from the study at any stage. The demographic information is provided in Table 1.

### 3.2. Instrumentation

#### 3.2.1. Exposure to Terrorism

The exposure to terrorism was measured using four items of the terrorism exposure scale adopted from Hobfoll, Canetti-Nisim, and Johnson [32]. The sample items are “I was present at the scene during a terrorist attack” and “My friend or family member was injured in a terrorist attack”. In addition, a 5-point Likert scale, where 1 is “strongly disagree” and 5 is “strongly agree”, was used to measure responses.

#### 3.2.2. Post-Traumatic Stress Disorder

For assessing PTSD, a shorter version with 10 items, Post Traumatic Stress Disorder Scale, was adopted from Boudewyns and Hyer [33]. The sample items are, “I cannot concentrate on my tasks” and “other things kept making me think about it”. In addition, a 5-point Likert scale, where 1 is “strongly disagree” and 5 is “strongly agree”, was used to measure responses.

#### 3.2.3. Social Support

The Multi-Dimensional Perceived Social Support Scale (revised form) was assessed using a 12-item scale adapted from Çevik and Yildiz [34]. The sample items are, “There is a special person who is around when I am in need” and “I can talk about my problems with my friends”. The scale contained four items focused on support from significant others, four on support from family members, and four on support from friends. A 5-point Likert scale, where 1 is “strongly disagree” and 5 is “strongly agree”, was used to measure responses.

#### 3.2.4. Employee Performance

The university employee performance was measured using a 10-item scale adapted from Griffin, Neal, and Parker [35]. The sample items are, “I carried out the core parts of your job well’ and “I ensured that the tasks were completed properly.” A 5-point Likert scale, where 1 is “strongly disagree” and 5 is “strongly agree”, was used to measure responses.

## 4. Results

### 4.1. Control Variables

To examine the control variables, we used a one-way ANOVA test. The results indicated that SS (F = 5.33, *p* = 0.02) and EP (F = 4.52, *p* = 0.04) were significantly different based on gender, and TE (F = 5.28, *p* = 0.006; F = 4.79, *p* = 0.009) was significantly different based on work experience and age, while the rest of the demographic variables had no significant effect on the study variables. Hence, we have controlled gender, work experience, and age while conducting further analysis.

### 4.2. Common Method Variance

We have used Herman’s single factor analysis to check the common method variance using exploratory factor analysis by employing principal component analysis with no rotation and all measured items loaded into a single factor. The single factor explained about 46% variance below the recommended threshold of 50% [36].

### 4.3. Non-Response Bias

For non-response bias, we compared the means of 89 early and 89 late responses using an independent sample *t*-test. Results showed no significant difference in the early and late response of all constructs (See Table 2). Hence, it was concluded that there was no issue of common method variance and non-response bias.

### 4.4. Validity and Reliability

The confirmatory factor analysis (CFA), based on the four-factor model, namely, SS, PTSD, TE, and EP, was used to assess the validity and reliability of the data. The CFA resulted in an acceptable fit (GFI = 0.92, CFI = 0.92, AGFI = 0.74, RMSEA = 0.06, χ2 = 978, df = 553 *p* < 0.001). The reliability and validity of the collected data were assured. All indicators loaded significantly (*p* < 0.001) except (EP10) on their respective constructs and provided evidence of convergent validity (see Table 3). The values of AVE were also higher than the cut-off value of 0.5. Similarly, the discriminant validity was assessed using the Fornell and Larcker [37] criterion, where the AVE values of all latent variables were greater than the shared variance of each factor (see Table 4). Data reliability was evident from the Cronbach alpha values (ranging from 0.84 to 0.96) and composite reliability (ranging from 0.85 to 0.96).

### 4.5. Hypotheses Testing

The proposed model is a moderated mediation model where social support (SS) acts as the second-stage moderator, PTSD is the mediator, and university teachers’ performance (EP) is the dependent factor. We tested our hypotheses in a sequence where we first estimated a simple mediation model using Model 4 PROCESS Macro [38]. Next, we estimated the moderated mediation model using Model 14 PROCESS Macro [38]. Finally, moderation effects were probed.

First, we used Process Model 4 to test a mediation model with TE as the independent variable, PTSD as the mediator, and EP as the dependent variable. The results of Process Model 4 are presented in Table 5.

The results of the direct effect show that TE is negatively associated with EP (b = −0.499, *p*-value = 0.000); thus, Hypothesis 1 is supported. The direct effect of TE on PTSD is also significant and positive (unstandardized b = 0.933, *p*-value = 0.000), thus supporting Hypothesis 2. Similarly, PTSD has a negative impact on EP (b = −0.206, *p*-value = 0.002); hence, Hypothesis 3 is supported. Finally, the results of mediation analysis show that PTSD partially mediates the relationship between TE and EP (b = −0.19, *p*-value = 0.00), supporting Hypothesis 4.

Then, we used Process Model 14 to test our full moderated mediation model. SS was modeled as a moderator on the effect of PTSD on EP. This step added the SS and PTSD interaction to the regression equation predicting EP, increasing the explained variance in EP. (ΔR2 = 0.04, ΔF (178) = 24.4, *p* = 0.00). In summary, TE leads to PTSD (b = 0.93, t = 178) = 14.16, *p* = 0.000, sr2 = 0.53), which has an insignificant negative impact on EP (b = −0.018, t (178) = −0.342, *p* = 0.732, sr2 = 0.69). SS significantly attenuates the negative relationship between PTSD and EP (b = 0.207, t (178) = 4.947, *p* = 0.00, sr2 = 0.69) such that higher values of SS reduce the negative impact of PTSD on EP. The impact of PTSD on EP is significant and negative at a lower value of SS and becomes significantly positive at higher values of SS. The direct effect of TE on EP is negative and significant (b = −0.205), while the indirect effects through PTSD at different values of SS are not significant. The impact becomes positive at a higher SS value but remains insignificant. The results of Process Model 14 are presented in Table 6 and Table 7.

**Figure 2:**
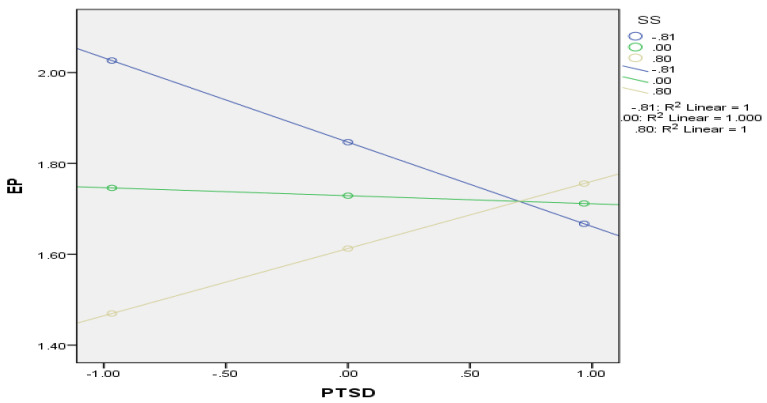
Interaction Plot.

In addition to the hypothesized relationships, we tested the conditional effect in the presence of a second-stage moderator. Table 4 shows that the magnitude of the conditional effect of PTSD on EP are significant at high and low values of SS. At high levels of PTSD, high SS significantly improves EP, while low SS will significantly decrease PE. We have plotted the interaction effects (see Figure 2) and found that PTSD and SS interaction significantly affect the PTSD and EP relationship.

## 5. Discussion and Conclusions

Firstly, this study examined that exposure to terrorism can significantly impact employee performance (Hypothesis 1). The results are consistent with the earlier studies [39]. According to Alpak and Ozcelik [39], employees who have been directly affected by terrorism or who have witnessed terrorist attacks are more likely to experience symptoms of post-traumatic stress disorder (PTSD). These mental health conditions can impair cognitive function and motivation, decreasing job performance and productivity. The previous findings come from different events with traumatic potential [2,13,40].

Furthermore, fear of future terrorist attacks can cause stress and anxiety, interfering with employees’ ability to concentrate and make decisions [3,41]. Therefore, it is essential for employers to be aware of the potential effects of terrorism on their employees and to provide support and resources to help mitigate these effects. This may include offering counseling services, flexible work arrangements, or time off for those directly affected by terrorism [10,41].

Bader and Berg [9] argued that exposure to terrorism significantly impacts employees’ performance, well-being, and mental health. Employers have a responsibility to provide support and resources to help employees cope with the effects of terrorism and to promote a safe and healthy work environment. Exposure to terrorism affects the performance of university teachers in several ways, such as by creating psychological distress. Exposure to terrorism can cause employees to experience psychological distress and PTSD. This can lead to decreased job performance, as workers with psychological distress are more likely to experience reduced productivity, absenteeism, and increased turnover [42]. At the same time, it decreases the concentration and memory of employees, which can result in decreased work performance [40]. It also causes workplace stress and worsens job satisfaction and performance [4,43]. Another aspect of exposure to terrorism is reduced motivation by inducing fear in employees, uncertainty, and loss of control, which can reduce motivation and lead to decreased work performance [9,44]. Above all, employees’ physical health is affected, leading to decreased work performance and increased absenteeism [40].

Secondly, this study examined the impact of exposure to terrorism on post-traumatic stress disorder (Hypothesis 2). The results of the present study support the findings of earlier studies [8,12]. Exposure to terrorism can have a significant impact on an individual’s mental health and can lead to the development of post-traumatic stress disorder. PTSD is a mental health condition that can develop after exposure to a traumatic event such as a terrorist attack. The symptoms of PTSD include re-experiencing the traumatic event through flashbacks or nightmares, avoidance of reminders of the event, negative changes in thoughts and feelings, and increased feelings of anxiety, irritability, and sleep disturbances. Research has shown that individuals directly or indirectly exposed to terrorism are at a higher risk of developing PTSD [11].

There are various causes of exposure to terrorism, including direct exposure to a terrorist attack, indirect exposure through media coverage, and vicarious exposure through hearing about the experiences of others [45]. The level of exposure and the individual’s personal, social, and cultural context can also play a role in determining the impact of exposure to terrorism on an individual’s mental health [2,13]. It is important to note that not all individuals who are exposed to terrorism will develop PTSD, and various factors can influence the likelihood of developing PTSD, including a history of mental illness, prior exposure to traumatic events, and social support [4,13]. So, exposure to terrorism can have a significant impact on an individual’s mental health and lead to the development of PTSD. Therefore, mental health professionals need to be aware of the causes of exposure to terrorism and its potential impact on an individual’s mental health [15].

Thirdly, the present study examined the impact of post-traumatic stress disorder on teachers’ performance (Hypothesis 3). Again, the results are aligned with the existing literature [8,16]. PTSD significantly impacts teachers’ performance and outcomes. A mental health disorder can develop after experiencing or witnessing a traumatic event, such as a natural disaster, violence, or abuse. Like other first responders, teachers are exposed to stressful events in their daily work, which can develop PTSD. Studies have shown that teachers with PTSD may experience intrusive thoughts, flashbacks, avoidance behaviors, and emotional numbness [17,46]. These symptoms can affect their ability to perform their job effectively, resulting in decreased job satisfaction, increased absenteeism, and higher levels of burnout. Additionally, teachers with PTSD may struggle to form strong relationships with their students and colleagues, which can further negatively impact their performance [47].

Fourthly, post-traumatic stress disorder (PTSD) is found to mediate the relationship between terrorism exposure and university teachers’ performance (Hypothesis 4). The existing literature supports the results, such as Wolmer, Hamiel, and Laor [48] and Smith and Jones [49]. Research has shown that the experience of terrorism exposure can significantly impact the mental health of university teachers, leading to increased symptoms of PTSD [48]. The resulting PTSD symptoms, such as intrusive memories, avoidance, and hyperarousal, can impair cognitive functioning, emotional regulation, and interpersonal skills, affecting their overall academic performance [49]. This highlights the importance of addressing the psychological well-being of university teachers who have been exposed to terrorism to mitigate the negative impact on their performance.

Despite these challenges, there are ways in which teachers with PTSD can receive support to manage their symptoms and improve their outcomes. For example, social support, mindfulness-based stress reduction, and exposure therapy effectively treat PTSD in teachers [12]. Additionally, universities can implement programs that promote mental health and support teachers who have experienced trauma. So, PTSD can have a significant impact on the performance and outcomes of teachers. However, with appropriate social support and treatment, teachers with PTSD can manage their symptoms and continue positively contributing to the education system.

Lastly, the question of whether social support moderates post-traumatic stress disorder and university employee performance was examined (Hypothesis 5). Social support is a widely recognized psychological treatment for post-traumatic stress disorder (PTSD). Soltesz et al. [50] found that PTSD people with inadequate support were likely to report difficulties in the workplace, including poor concentration, absenteeism, and decreased job satisfaction. It is important to note that these studies demonstrate a correlation between the lack of availability of support from their organization and decreased work performance in individuals with PTSD, and that they do not establish causality. Post-traumatic stress disorder (PTSD) significantly impacts teachers’ mental health and job performance, potentially leading to burnout and reduced effectiveness in the classroom. However, research suggests that social support can help to mitigate the adverse effects of PTSD on teacher performance. For example, Liu et al. [28] found that social support from colleagues and supervisors was associated with lower levels of burnout and higher job satisfaction among teachers with PTSD. Another study found that social support from family and friends was associated with lower PTSD symptoms and higher levels of job performance among teachers [12,51].

Teachers with social support were better able to cope with the challenges of their job and may be more resilient in the face of trauma and stress. However, it is important to note that not all forms of social support are equally effective. For example, a study by Kim et al. [51] found that colleague support was more strongly associated with job performance than support from family and friends. This suggests that social support within the workplace may be particularly important for teachers’ performance.

Social support can play an important role in mitigating the adverse effects of PTSD on teachers’ job performance. Teachers who have access to supportive colleagues and supervisors may be better able to cope with the challenges of their job and may be more effective in the classroom. Social support can act as a protective factor against the negative psychological impact of traumatic events. Individuals with high levels of social support are more likely to receive emotional validation, understanding, and assistance in coping with traumatic experiences. This emotional buffering can mitigate the development and severity of PTSD symptoms [45]. In addition, having a strong social support network fosters a sense of belonging and social integration, which are important factors in post-trauma recovery. High levels of social support can reduce feelings of isolation and alienation that often accompany traumatic experiences. This sense of belonging can promote resilience and facilitate healing [25].

Moreover, social support provides individuals with tangible assistance, such as practical help or resources. Having access to high levels of instrumental support can equip individuals with the necessary tools to cope with the aftermath of traumatic events. This may include financial assistance, access to healthcare services, or guidance in navigating legal or bureaucratic procedures. In addition, these resources can alleviate stressors and contribute to better mental health outcomes [32].

## 6. Practical and Theoretical Implications

Exposure to terrorism has significant implications for individuals, communities, and even nations. It can cause physical harm, death, and property loss, leaving long-lasting psychological scars. One of the most common mental health outcomes of exposure to terrorism is post-traumatic stress disorder (PTSD). PTSD is a condition that results from experiencing or witnessing a traumatic event, such as a terrorist attack. It can cause severe symptoms, such as recurrent memories, avoidance behaviors, and increased anxiety and depression.

Teachers play a crucial role in shaping students’ lives, and exposure to terrorism can harm their performance. Teachers who have experienced or witnessed a traumatic event may experience symptoms of PTSD, which can affect their ability to perform their duties effectively. They may find it challenging to focus, exhibit irritability, and experience difficulty forming meaningful relationships with students, which can harm their teaching performance.

Teachers who have experienced or witnessed a traumatic event can experience symptoms of PTSD, which can affect their ability to perform their duties effectively. However, social support as a moderator can help to mitigate the adverse effects of PTSD and improve teachers’ performance. By providing teachers with the tools they need to manage their symptoms, increase their resilience, and develop coping strategies, social support can help them continue performing their duties effectively, even in the face of traumatic events.

Social support is an influential factor for PTSD and helps teachers overcome the symptoms they experience following exposure to terrorism. Social support is a crucial element in recovery for individuals who have experienced traumatic events. Social support can play a critical role in reducing the severity of PTSD symptoms and improving the overall mental health of those affected. In addition, social support can help mitigate traumatic experiences’ negative impact on mental health. Social support can come in various forms: emotional, practical, informational, and companionship. In addition, emotional support can be highly beneficial in reducing symptoms of PTSD, such as anxiety, depression, and irritability. Emotional support can provide a sense of safety, security, and comfort to individuals who have experienced trauma, which can help them to manage their symptoms and promote healing.

In addition to reducing PTSD symptoms, social support can also enhance teachers’ performance in the workplace. Teachers play a crucial role in the education and development of children, and their performance can significantly impact their students’ academic outcomes. Social support gives teachers the resources and encouragement they need to perform at their best. Supportive colleagues and supervisors can help teachers feel valued, respected, and motivated, increasing job satisfaction and better performance. Social support is valuable in promoting mental health and enhancing performance in various settings. By providing emotional, practical, and informational support, social support can help mitigate traumatic events’ adverse effects and promote healing. Additionally, social support can enhance teachers’ performance by providing them with the resources and encouragement they need to perform at their best. It is essential to recognize the importance of social support in these contexts and to work to cultivate supportive environments that promote healing and success.

The theoretical implications of the relationship between exposure to terrorism, post-traumatic stress disorder (PTSD), and university teachers’ performance are significant. The findings suggest that terrorism exposure can have detrimental effects on the performance of university teachers, which the development of PTSD symptoms may mediate. This supports the understanding that exposure to traumatic events, such as terrorism, can have lasting impacts on individuals’ mental health and subsequent work performance. Moreover, identifying social support as a potential boundary condition highlights the importance of supportive environments in attenuating the negative effects of PTSD on employee performance. These findings contribute to existing theories on trauma, PTSD, and the role of social support in the workplace, providing insights for further research and practical implications for organizational policies and practice.

## 7. Limitations and Future Directions

Research limitations for this study include limited data from the conflict-affected region, difficulties in accurately measuring exposure and PTSD symptoms, and the lack of control for confounding variables such as prior trauma exposure and personality characteristics. Regarding teachers’ performance, limitations include a limited understanding of the specific mechanisms through which exposure to terrorism affects teacher performance and the need to further examine the potential moderating effects of social support. Future directions include incorporating more diverse populations and expanding the investigation of the impact of exposure to terrorism on teacher well-being, job satisfaction, and work-life balance, as well as exploring other potential moderators such as resilience and behavioral therapies. Similarly, future research can focus on the differential effects of different types of social support (i.e., family and friends) on trauma and stress.

## Figures and Tables

**Table 1:** Demographic Information of Respondents, n = 178.

Variable	Category	No. of Respondents	Percentage
Age	26–35 years	41	23
	36–45 years	108	60.7
	46–60 years	29	16.3
	Total	178	100
Experience	1–5 years	127	71.3
	6–10 years	29	16.3
	Above 11 years	22	12.4
	Total	178	100
Gender	Male	115	64.6
	Female	63	35.4
	Total	178	100
Education	Bachelors	29	16
	Masters/MS	119	66.9
	PhD	30	16.9
	Total	178	100

Source: Data recorded from university teachers.

**Table 2:** Non-Response Bias Check.

Variable	Mean Difference	*t*-Value	df	*p*-Value
Age	−0.135	−1.44	176	0.151
Gender	0.056	0.78	176	0.436
Qualification	0.090	−1.01	176	0.312
Experience	−0.034	−0.32	176	0.749

**Table 3:** Results of Confirmatory Factor Analysis (CFA).

Construct/Variable	b	Alpha	CR	AVE
Social Support		0.94	0.93	0.60
SS 1	0.779			
SS 2	0.700			
SS 3	0.774			
SS 4	0.765			
SS 5	0.824			
SS 6	0.745			
SS 7	0.717			
SS 8	0.781			
SS 9	0.837			
SS 10	0.730			
SS 11	0.776			
SS 12	0.700			
PTSD		0.96	0.96	0.72
PTSD 1	0.892			
PTSD 2	0.853			
PTSD 3	0.817			
PTSD 4	0.850			
PTSD 5	0.791			
PTSD 6	0.851			
PTSD 7	0.863			
PTSD 8	0.829			
PTSD 9	0.882			
PTSD 10	0.859			
Terrorism Exposure		0.84	0.85	0.59
TE 1	0.750			
TE 2	0.730			
TE 3	0.769			
TE 4	0.794			
Employee Performance		0.91	0.92	0.57
EP 1	0.816			
EP 2	0.752			
EP 3	0.752			
EP 4	0.738			
EP 5	0.700			
EP 6	0.712			
EP 7	0.681			
EP 8	0.748			
EP 9	0.819			
Goodness of fit Indices				
χ^2^ = 978; d.f. = 553; χ^2^/d.f. = 1.77; *p* < 0.001; CFI = 0.92; GFI = 0.78; AGFI = 0.74; RMR = 0.04; RMSEA = 0.06				

b: Standardized coefficient; Alpha: Cronbath’s Alpha; CR: Composite Reliability; AVE: Average Variance Extracted.

**Table 4:** Descriptive Statistics and Correlations.

	Variable	No of Items	Mean	s.d.	TE	PTSD	SS	EP
1	TE	4	3.98	0.76	0.59			
2	PTSD	10	3.97	0.96	0.730 ** (0.53)	0.72		
4	SS	12	4.11	0.81	0.744 ** (0.55)	0.751 ** (0.56)	0.60	
5	EP	9	1.85	0.72	−0.726 ** (0.52)	−0.659 ** (0.43)	0.741 ** (0.54)	0.57

** Correlation significant at 0.01; Shared Variance are in parenthesis; AVE is on diagonal; TE = Terrorism Exposure; PTSD = Post-Traumatic Stress Disorder; EP = Employee Performance.

**Table 5:** 5000 Bootstrap Results for Direct and Indirect Effects PROCESS Model 4.

Path	Estimate	SE	LL 95% CI	UL 95% CI
TE → EP (Direct Effect)	−0.499 *	0.06	−0.636	−0.361
PTSD → EP	−0.206 *	0.05	−0.314	−0.099
Standardized Indirect Effects using 5000 Bootstrap 95% CI				
Path	Effect	SE	LL 95% CI	UL 95% CI
TE → PTSD → EP	−0.192 *	0.06	−0.348	−0.078

TE = Terrorism Exposure; PTSD = Post-Traumatic Stress Disorder; EP = Employee Performance. * *p* < 0.01.

**Table 6:** 5000 Bootstrap Moderation Results for PROCESS Model No 14.

	DV: SP			
	Estimate	SE	LL 95% CI	UL 95% CI
TE	−0.2052 *	0.068	−0.339	−0.071
PTSD	−0.0177	0.052	−0.120	0.084
SS	−0.1451	0.086	−0.315	0.025
PTSD*SS	0.2068 **	0.042	−0.186	−0.018
Model Fit				
F-value	97.44 *			
R^2^	0.69			
R^2^ Change	0.04 *			
Conditional Effects of PTSD at values of SS using 5000 Bootstrap 95% CI				
Conditional effect at different values of SS	Effect	SE	LL 95% CI	UL 95% CI
−0.8126	−0.186 *	0.06	−0.302	−0.069
0.000	−0.018	0.05	−0.120	0.084
0.8015	0.148 *	0.06	0.021	0.275

TE = Terrorism Exposure; PTSD = Post-Traumatic Stress Disorder; EP = Employee Performance. * *p* < 0.01, ** *p* < 0.05.

**Table 7:** 5000 Bootstrap Moderation Results for PROCESS Model No 14.

Standardized Direct Effects Using 5000 Bootstrap 95% CI TE → EP				
Effect	SE	LL 95% CI	UL 95% CI	
−0.205 *	0.07	−0.339	−0.071	
**Conditional Indirect Effects Using 5000 Bootstrap 95% CI** **TE → PTSD → EP**				
Indirect effect at different values of SS	Effect	SE	LL 95% CI	UL 95% CI
−0.8126	−0.173	0.10	−0.403	0.024
0.000	−0.016	0.09	−0.149	0.204
0.8015	0.138	0.11	−0.028	0.412

TE = Terrorism Exposure; PTSD = Post-Traumatic Stress Disorder; EP = Employee Performance; * *p* < 0.01.

## Data Availability

The data will be available on request from the corresponding author.
